# Protective Effects of One 2,4-Dihydro-3H-Pyrazol-3-one Derivative against Posterior Capsular Opacification by Regulation of TGF-β2/SMADs and Non-SMAD Signaling, Collagen I, and Fibronectin Proteins

**DOI:** 10.3390/cimb44100343

**Published:** 2022-10-19

**Authors:** Chun-Ching Shih, Chia-Yi Lee, Fung-Fuh Wong, Cheng-Hsiu Lin

**Affiliations:** 1 Department of Nursing, College of Nursing, Central Taiwan University of Science and Technology, No.666 Buzih Road, Beitun District, Taichung City 40601, Taiwan; 2 Institute of Medicine, Chung Shan Medical University, Taichung City 40201, Taiwan; 3 Nobel Eye Institute, Taipei 100008, Taiwan; 4 Department of Ophthalmology, Jen-Ai Hospital Dali Branch, Taichung City 412224, Taiwan; 5 School of Pharmacy, China Medical University, No. 91 Hsueh-Shih Road, Taichung City 40402, Taiwan; 6 Department of Internal Medicine, Fengyuan Hospital, Ministry of Health and Welfare, Fengyuan District, Taichung City 42055, Taiwan

**Keywords:** cataract, posterior capsule opacification, Sma and mad protein (SMAD)s pathway, non-SMADs pathway, transforming growth factor type β2 (TGF-β2

## Abstract

Many elderly individuals frequently experience cataracts that interfere with vision. After cataract surgery, the left lens epithelial cell (LEC) exhibited fibrosis and posterior capsule opacification (PCO). Sometimes, there is a need for a second surgery; nevertheless, people try other methods, such as a good pharmacological agent, to treat PCO to reduce transforming growth factor-β2 (TGF-β2) amounts to avoid secondary surgery. The aim of the present study was to explore the potential anti-PCO activity of five 2,4-dihydro-3H-pyrazol-3-one (DHPO) derivatives in a TGF-β2-induced fibrogenesis SRA01/04 cell model. The 2-phenyl-5-propyl-DHPO (TSE; no. 2: TSE-2) compound showed the best activity of reduced expression levels of TGF-β2 among five derivatives and therefore was chosen to evaluate the anti-PCO activity and molecular mechanisms on the Sma and mad protein (SMAD) signaling pathway (including TGF-β2, SMADs, and the inhibition of nuclear translocation of SMADs), non-SMAD pathway proteins, including p-extracellular, regulated protein kinases (ERK) 1/2, or *p*-c-Jun N-terminal kinase (JUN) by Western blotting, PCR, or confocal immunofluorescence analyses. Following treatment with 10 μg/mL of the five compounds, the cells displayed great viability by 3-(4,5-dimethylthiazol-2-yl)-5-(3-carboxymethoxyphenyl)-2-(4-sulfophenyl)-2H-tetrazolium (MTT) assay. In this study, the result of lactate dehydrogenase (LDH) activity measurement did not affect the cytotoxicity of the five compounds. In TGF-β2-induced fibrogenesis in SRA01/04 cells, treatment with the TSE compound decreased the TGF-β2/SMAD signaling genes, including reduced mRNA or expression levels of TGF-β2, SMAD3, and SMAD4, leading to inhibition of TGF-β2-induced fibrogenesis. Our confocal immunofluorescence analyses demonstrated that TSE treatment displays a suppressive effect on SMAD2/3 or SMAD4 translocation to the nucleus. Furthermore, TSE treatment exhibits a reduction in the non-SMAD target gene expression levels of *p*- c-Jun N-terminal kinase (JUN), *p*- extracellular, regulated protein kinases (ERK)1/2, *p*- p38 mitogen-activated protein kinase (p38), *p*-phosphatidylinositol 3-kinase (PI3K), *p*-mammalian target of rapamycin complex (mTORC), *p*-Akt (Ser^473^), and *p*-Akt (Thr^308^). The overall effect of TSE is to reduce the expression levels of collagen I and fibrinogen (FN), thus contributing to antifibrotic effects in cell models mimicking PCO. Our findings reveal the benefits of TSE by regulating TGF-β/SMAD signaling and non-SMAD signaling-related gene proteins to display antifibrotic activity in cells for the possibility of preventing PCO after cataract surgery.

## 1. Introduction

Cataracts frequently occur in elderly individuals. Posterior capsular opacification (PCO) is an ocular complication disease after cataract surgery, and the principal reasons are fibrosis [1] of the entire posterior and left anterior capsule with lens epithelial cells (LECs) adhered [2,3]. These quickly develop and finally interfere with the visual axis for the next visual deprivation [2,3,4] after cataract surgery. Finally, once there is worse enough inhibition of visual quality, another surgery must be performed [2,3].

There are various means in the management and prevention of PCO, including appropriate anti-inflammatory drugs [5], anti-metabolic agents (such as 5-fluorouracil, mitomycin) [6,7], improved intraocular lens (IOL) designs (such as different materials, construction and designs to create a barrier to retard cell growth on the posterior capsule) [8,9], good surgical advances, drug delivery systems (such as a closed bag drug delivery system), and biological targets [1]. The design of IOLs and improving surgical skills and materials result in the amelioration of PCO [2,3] but have not eradicated the problem [10], and trauma and toxic side effects (such as releasing drugs toward other tissues that are harmful to the corneal endothelium) exist [11]. Numerous growth factors function in certain signaling pathways; thus, therapeutic intervention is thought to be via amelioration or regulation of crucial signaling targets [2,3].

Transforming growth factor-β2 (TGF-β2) signaling has been shown to play an important role in PCO growth following cataract surgery [1] Accumulating evidence reveals that TGF-β2 plays a core role in the regulation of LEC behavior during lens repair after injury [1,2,3,12] and cataract surgery [3,13]. Therefore, previous evidence has shown that strategies attenuating TGF-β2 signaling could be efficacious in the inhibition of capsular fibrosis [10] or PCO after cataract surgery.

Furthermore, TGF-β2 has been identified to activate not only its SMAD signaling pathway downregulation but also the constitution of PCO [14]. Saika et al. and other researchers demonstrated that SMADs are targets associated with intracellular signal transduction from cell exterior TGF-β receptors to nuclear gene promoters [3,13,15], and once ligands bind with the TGF-β receptor, translocation of a complex of phosphorylated SMAD2 and 3 occurs to the nuclei in a complex with SMAD4 and sequela [13]. The TGF-β family is a representative epithelial–mesenchymal transition (EMT) inducer in both development and disease through SMAD- and non-SMAD-regulated pathways [15,16]. Non-SMAD signaling pathways induce epithelial–mesenchymal transition (EMT) with translation via the PI3K/AKT/mTOR pathway and promote cytoskeletal remodeling [17].

**Figure 1:**
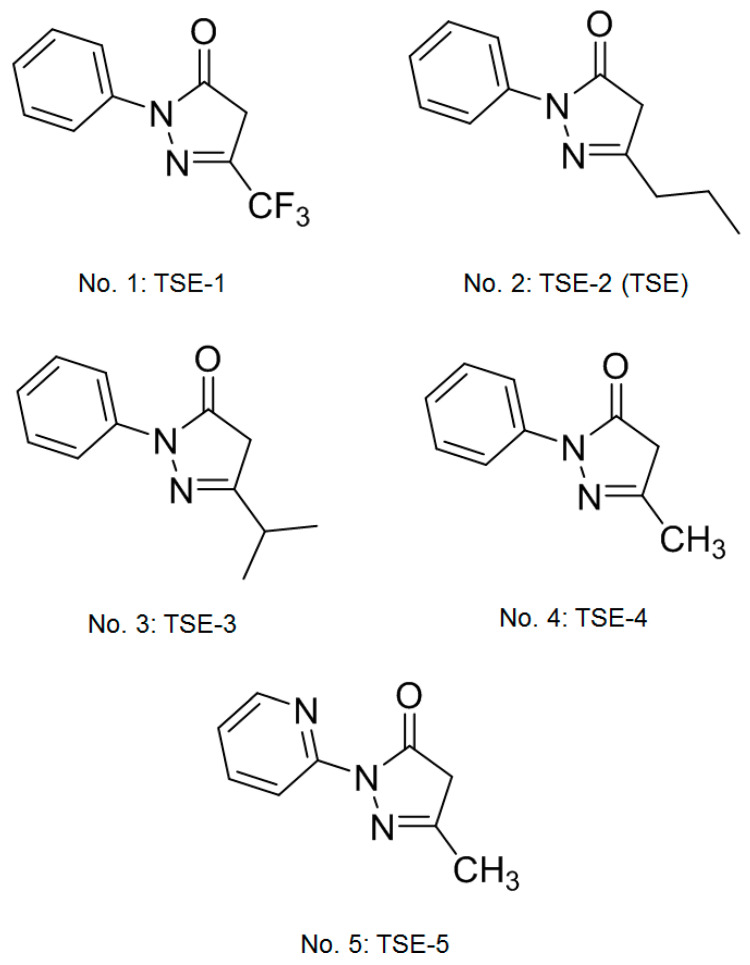
The chemical structure of five compounds of 2,4-dihydro-3H-pyrazol-3-one (DHPO) derivatives [19]. No. 1: TSE-1: 2-phenyl-5-(trifluoromethyl)-2,4-dihydro-3H-pyrazol-3-one, chemical formula: C_10_H_7_F_3_N_2_O. No. 2: TSE-2 (TSE): 2-phenyl-5-propyl-2,4-dihydro-3H-pyrazol-3-one, chemical formula: C_12_H_14_N_2_O. No. 3: TSE-3: 5-isopropyl-2-phenyl-2,4-dihydro-3H-pyrazol-3-one, chemical formula: C_12_H_14_N_2_O. No. 4: TSE-4. 5-methyl-2-phenyl-2,4-dihydro-3H-pyrazol-3-one, chemical formula: C_10_H_10_N_2_O. No. 5: TSE-5: 5-methyl-2-(pyridin-2-yl)-2,4-dihydro-3H-pyrazol-3-one, chemical formula: C_9_H_9_N_3_O.

However, there is a lack of effective pharmacological therapies without toxicity for the prevention of PCO. Pirfenidone (5-methyl-1-phenyl-2-[1H]-pyridone; PFD) has been demonstrated to be an anti-inflammatory drug with anti-fibrosis activity, and it has been used in clinical agents for the management of idiopathic pulmonary fibrosis (IPF) [18]. The structure of 2-phenyl-5-propyl-2,4-dihydro-3H-pyrazol-3-one (TSE) has a core structure similar to that of pirfenidone. In this study, we assessed whether TSE could have anti-PCO activity. First, we screened five compounds (no. 1: 2-phenyl-5-(trifluoromethyl)-DHPO, no. 2: 2-phenyl-5-propyl-DHPO (TSE; TSE-2), no. 3: 5-isopropyl-2-phenyl-DHPO, no. 4: 5-methyl-2-phenyl-DHPO, and no. 5: 5-methyl-2-(pyridin-2-yl)-DHPO) (Figure 1) [19], and our findings showed that TSE possessed the best activity in SRA01/04 cells. Nevertheless, the entire potential activities of TSE on PCO remain unknown in TFG-β2-induced PCO of the human LEC line SRA01/04. Thus, we evaluated whether TSE treatment could be effective against fibrosis, and it displayed better efficacy than PFD without toxic effects. Furthermore, the anti-PCO activity of TSE was assessed in TGF-β2-treated cells by modulating numerous targeted genes involved in SMAD or non-SMAD signaling pathways.

## 2. Materials and Methods

### 2.1. Chemicals

Antibodies against TGFβ-2 (no. ab36495), rabbit anti-SMAD3 antibody (no. ab84177), rabbit anti-SMAD4 antibody (no. ab236321), rabbit anti-β actin antibody (no. ab8227), anti-fibronectin antibody (Fn-3) (no. ab18265), and anti-collagen I (no. ab34710) were purchased from Abcam, Inc. Antibodies against phospho-p44/42 MAPK (ERK1/2) (Thr^202^/Tyr^204^) (no. 9101), p44/42 MAPK (ERK1/2) (no. 9102), phospho-SAPK/JNK (Thr^183^/Tyr^185^) (no. 9251), SAPK/JNK (no. 9252), phospho-p38 MAPK (Thr^180^/Tyr^182^) (28B10), mouse mAb (no. 9216), p38 MAPK (no. 9212), phospho-PI3 kinase p85 (Tyr^458^)/p55 (Tyr^199^) (no. 4228), PI3 kinase p85 (no. 4292), phospho-mTOR (Ser^2481^) (no. 2974), mTOR (no. 2972), phospho-Akt (Ser^473^) (no. 9271), phospho-Akt (Thr^308^) (no. 9275), and Akt (no. 9272) were purchased from Cell Signaling Technology, Inc., Danvers MA, USA. The secondary anti-rabbit antibody was from Jackson Lab., Inc. (West Grove, PA, West Baltimore Pike, USA).

### 2.2. Cell Lines

SRA01/04 cells (RCB1591), an SV40 T-antigen-transformed human LEC line, SV40 T-antigen-transformed human lens epithelial cell line [20], were purchased from Cell Bank Riken BioResource Research Center, Japan. The cells were cultured in media specific to the cell line, and this process was performed as described in a previous study [21].

### 2.3. MTT Assay

The 3-(4,5-dimethylthiazol-2-yl)-5-(3-carboxymethoxyphenyl)-2-(4-sulfophenyl)-2H-tetrazolium (MTT) measurement was used to evaluate cytotoxicity. SAR01/04 cells were seeded (1 × 10^4^ cells/mL) in 96-well culture plates and cultured with five compounds of DHPO derivatives at different concentrations (5, 10, or 20 mg/mL) for 24 h as described in a previous study [22].

### 2.4. Lactate Dehydrogenase (LDH) Activity Assay

After treatment with these five compounds, this part was performed as described in a previous study [23].

### 2.5. Immunoblotting Analysis

SRA01/04 cell lysates were lysed in radioimmunoprecipitation assay (RIPA) buffer, and the immunoblotting analysis was performed as described in previous studies [21,24] with the following specific antibodies: anti-TGFβ2 and anti-SMADs (Cell Signaling, Beverly, MA, USA).

### 2.6. Confocal Immunofluorescence Microscopy

Cells were seeded at a density of 2 × 10^5^ cells/well in 6-well plates as described in a previous study [25].

### 2.7. Relative Quantification of mRNA

The relative quantification of mRNA was performed as described in a previous study [24,26]. The primer sequences were as follows: TGFβ-2 primer: forward primer 5′- TGAAGTTCTAGCCATGAGGT -3′ reverse primer 5′- AGCAATTATCCTGCACATTT -3′; SMAD3 primers: forward primer 5′- TGAAGTTCTAGCCATGAGGT -3′ reverse primer 5′- AATATTTGGTTCCTGGGTCT -3′; SMAD4 primers: forward primer 5′- TAAGGCCATTTGTTTTGTTT-3′ reverse primer 5′- AGCCATTACTTTCAGGTTGA -3′; and β-actin primers: forward primer 5′- GGCGGACTATGACTTAGTTG -3′ reverse primer 5′-TGCCAATCTCATCTTGTTT-G-3′. The relative gene expression was assayed with agarose gel electrophoresis.

### 2.8. Statistical Analyses

Data are the mean and standard error. All results were analyzed with analysis of variance with Dunnett’s multiple range test by SPSS software. *p* < 0.05 was considered statistically significant.

## 3. Results

### 3.1. Effects of Five Compounds on the Expressions of TGF-β2, SMAD3, or SMAD4 in SRA01/04 Cells

**Figure 2:**
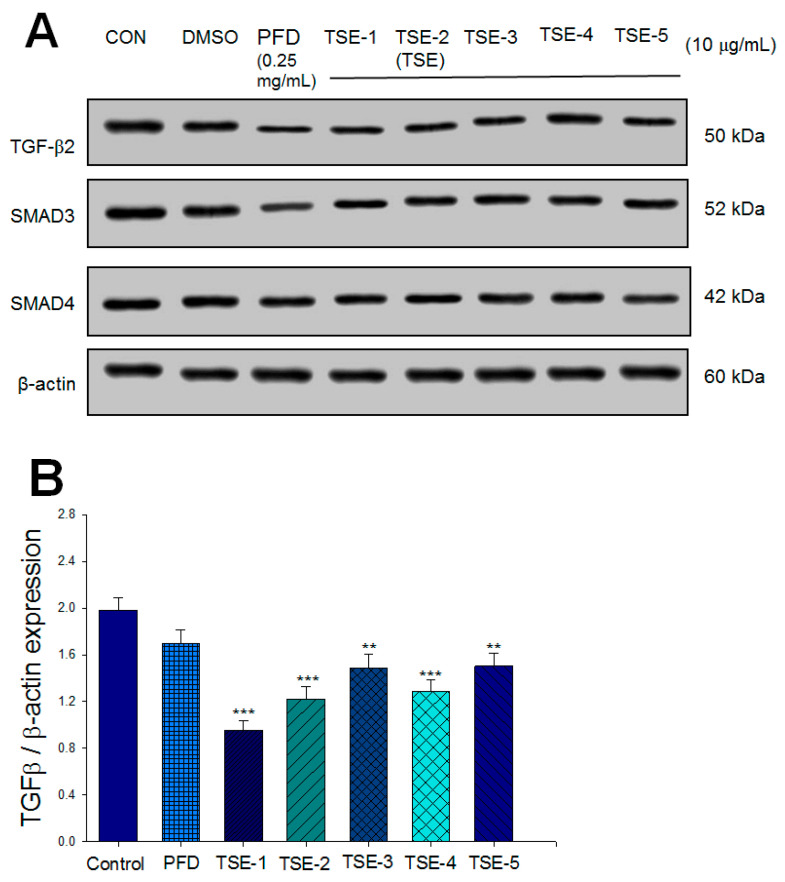

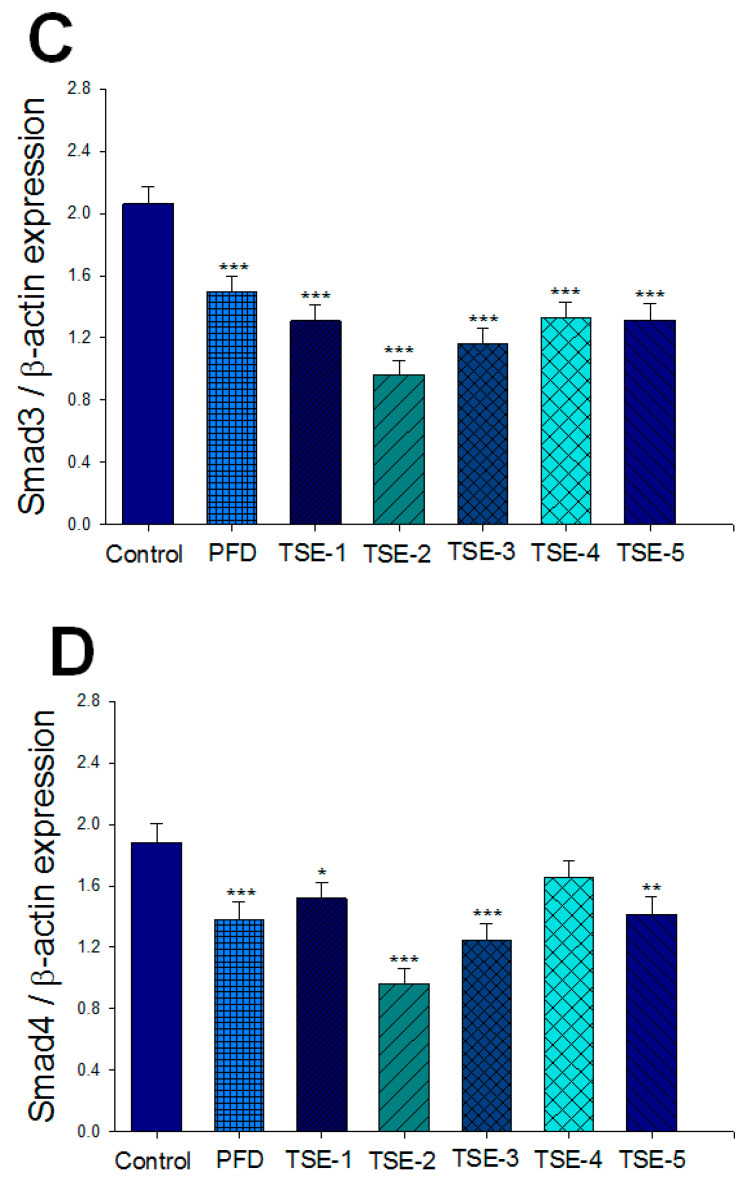
Screening tests of five compounds (including 2,4-dihydro-3H-pyrazol-3-one (DHPO) derivatives) in the expression levels of TGFβ2, SMAD3, or SMAD4 in cells using Western blot. (**A**) Representative image; quantification of (**B**) TGFβ2, (**C**) SMAD3, or (**D**) SMAD4 to β-actin. Protein was separated by 12% SDS-PAGE detected by Western blot. * *p* < 0.05, ** *p* < 0.01 or *** *p* < 0.001 compared to CON cells. Data are the means ±SE (n = 3).

There was no significant difference in the protein expression levels of TGF-β2, SMAD3, and SMAD4 between the DMSO group and the CON group. There was a significant decrease in the expression levels of TGF-β2 in the TSE-1-, TSE-2 (TSE)-, TSE-3-, TSE4-, and TSE-5-treated groups compared to the CON group (*p* < 0.001, *p* < 0.001, *p* < 0.01, *p* < 0.001, *p* < 0.01, respectively) (Figure 2A,B). There was a significant decrease in the expression levels of SMAD3 in the PFD-, TSE-1-, TSE-2 (TSE)-, TSE-3-, TSE4-, and TSE-5-treated groups compared to the CON group (*p* < 0.001, *p* < 0.001, *p* < 0.001, *p* < 0.001, *p* < 0.001, *p* < 0.001, respectively) (Figure 2A,C). There was a significant decrease in the expression levels of SMAD4 in the PFD-, TSE-1-, TSE-2 (TSE)-, TSE-3-, and TSE-5-treated groups compared to the CON group (*p* < 0.001, *p* < 0.05, *p* < 0.001, *p* < 0.001, *p* < 0.01, respectively) (Figure 2A,D). We found that TSE-2 (TSE) displayed a significant decrease in TGF-β2 expression compared with that in the CON group.

### 3.2. Cell Viability of Five Compounds

**Figure 3:**
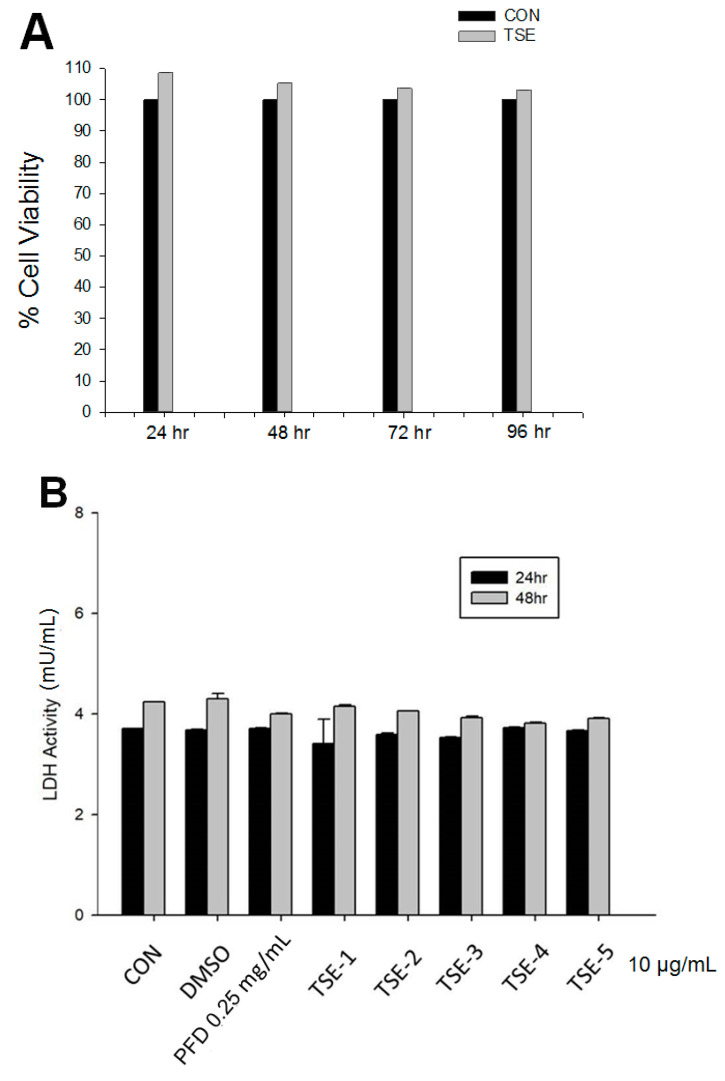
Cell viability and cytotoxicity. (**A**) Cell viability of 2-phenyl-5-propyl- DHPO (TSE; TSE-2). Following treatment with TSE at 24, 48, 72, or 96 h time, there was no significant difference between TSE treated and the CON. Data are the mean ±SE (n = 3). (**B**) Cytotoxicity of five compounds of DHPO derivatives. Following treatment with five compounds at 24 or 48 h time, no significant difference was found between five compounds treated and the CON group. Values are the mean ± SE (n = 3).

In the trypan blue exclusion test, after 24, 48, 72, or 96 h of treatment with five compounds of DHPO, the percentages of living cells were 108.6 ± 2.7%, 105.3 ± 1.4%, 103.6 ± 2.1%, and 103.2 ± 2.4% for the control and 10 μg/mL TSE groups, respectively (Figure 3A). No significant difference was found between the groups (*p* > 0.05).

### 3.3. LDH Assay

Following treatment with five compounds 24 h later, the cell-mediated lysis percentages were 4.47 ± 0.9% for the control and 10 μg/mL TSE groups and 7.36 ± 1.1% for the control and 10 μg/mL TSE groups 48 h later. TSE did not display significant cytotoxicity action (Figure 3B).

### 3.4. Effects of TSE on the mRNA Levels of Targeted Genes in Cells

**Figure 4:**
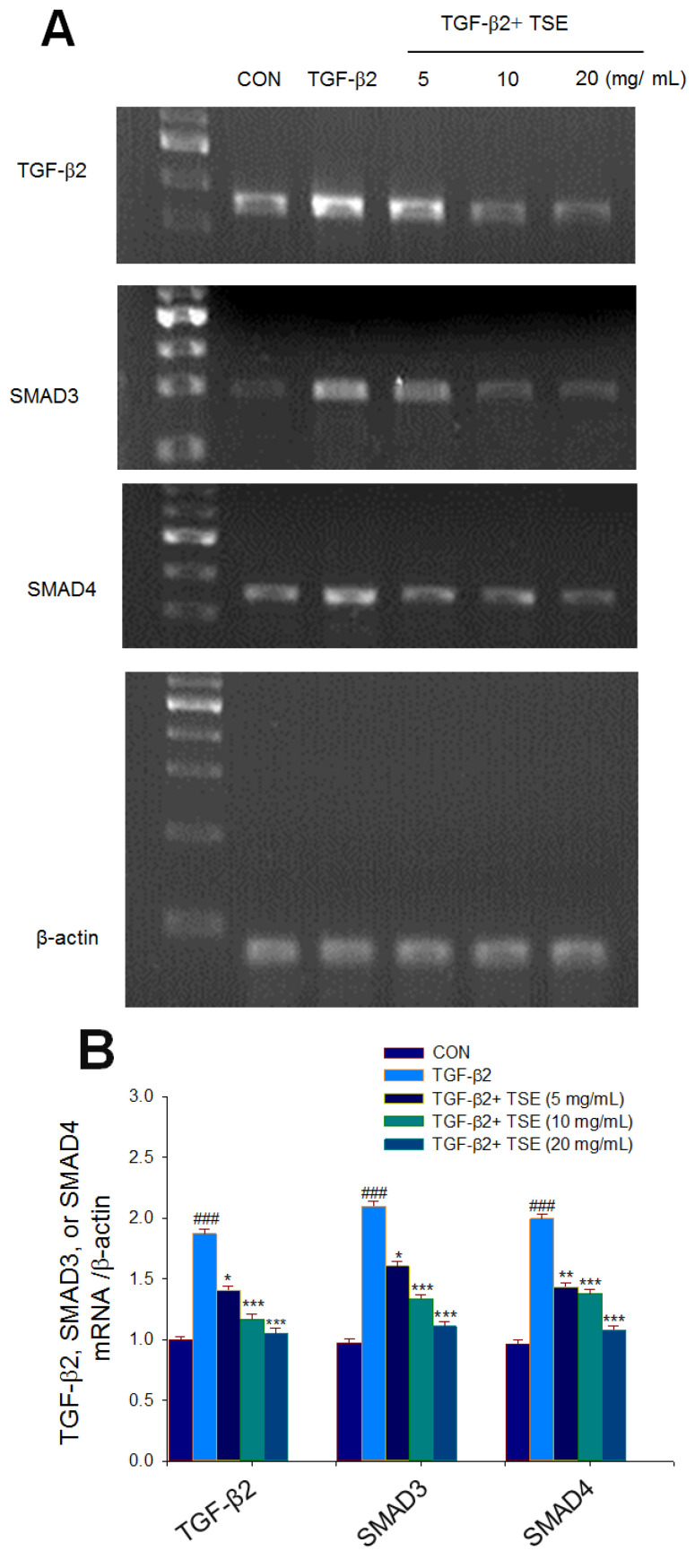

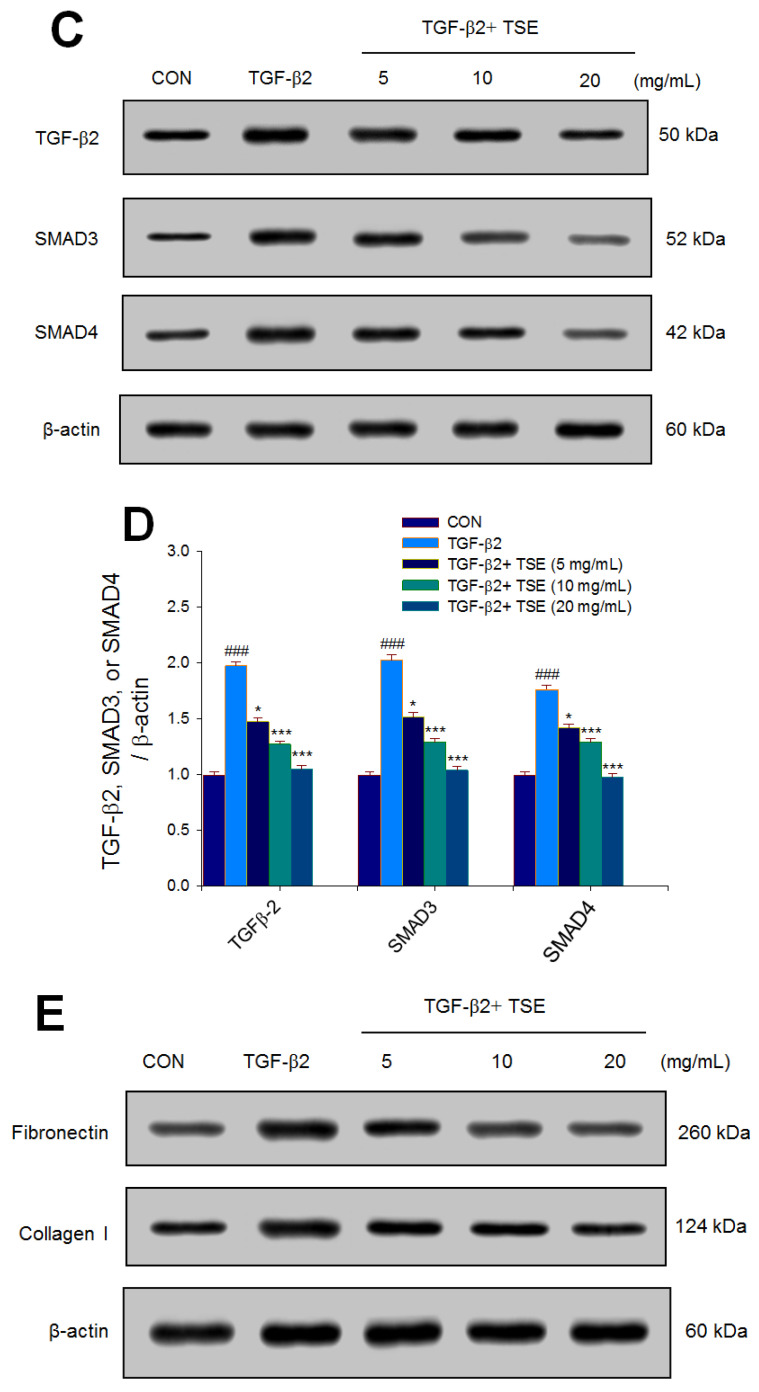

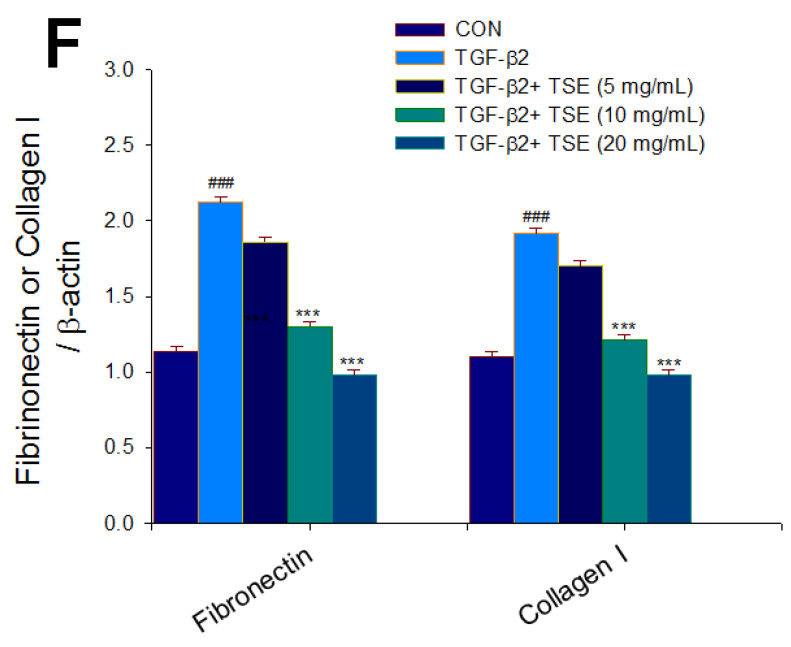
Effects of TSE on the mRNA or protein expression levels of TGFβ2, SMAD3, SMAD4, fibronectin, or collagen I in TGFβ2-induced SRA01/04 cells. (**A**) Representative image and (**B**) quantification of the ratio of target gene to β-actin mRNA levels. The RT-PCR amplification was performed on PCR Detection System. The relative gene expression is assayed with agarose gel electrophoresis. Protein was separated by 12% SDS-PAGE detected by Western blot: (**C**, **E**) representative image, and (**D**, **F**) quantification of the target gene to β-actin. ^###^ *p* < 0.001 in comparison to control cells; * *p* < 0.05, ** *p* < 0.01 or *** *p* < 0.001 in comparison to TGF-β2-induced control cells. Values are the mean ± SE (n = 3).

The mRNA levels of TGF-β2, SMAD3, and SMAD4 were markedly enhanced in SRA01/04 cells compared to the CON group (*p* < 0.001, *p* < 0.001, *p* < 0.001, respectively). A reduction in the mRNA levels of TGF-β2 was observed in the 5, 10, and 20 mg/mL TSE-treated groups compared to the vehicle-treated TGF-β2 group (*p* < 0.05, *p* < 0.001, *p* < 0.001, respectively) (Figure 4A,B). A reduction in the mRNA levels of SMAD3 was observed in the 5, 10, and 20 mg/mL TSE-treated groups compared to the vehicle-treated TGF-β2 group (*p* < 0.05, *p* < 0.001, *p* < 0.001, respectively) (Figure 4A,B). A reduction in the mRNA level of SMAD4 was observed in the 5, 10, and 20 mg/mL TSE-treated groups compared to the vehicle-treated TGF-β2 group (*p* < 0.01, *p* < 0.001, *p* < 0.001, respectively) (Figure 4A,B).

### 3.5. Effects of TSE on the Expression Levels of TGF-β2, SMADs, Fibronectin, and Collagen I

The protein expression levels of TGF-β2, SMAD3, SMAD4, fibronectin, and collagen I were significantly increased in TGF-β2-induced cells compared to the CON group (*p* < 0.001, *p* < 0.001, *p* < 0.001, *p* < 0.001, *p* < 0.001, respectively) (Figure 4C–F). Decreases in the expression levels of TGF-β2 were observed in the 5, 10, and 20 mg/mL TSE-treated groups compared with the vehicle-treated TGF-β2 group (*p* < 0.05, *p* < 0.001, *p* < 0.001, respectively) (Figure 4C,D). Decreases in the expression levels of SMAD3 were observed in the 5, 10, and 20 mg/mL TSE-treated groups compared with the vehicle-treated TGF-β2 group (*p* < 0.05, *p* < 0.001, *p* < 0.001, respectively) (Figure 4C,D). Decreases in the expression levels of SMAD4 were observed in the 5, 10, and 20 mg/mL TSE-treated groups compared with the vehicle-treated TGF-β2 group (*p* < 0.05, *p* < 0.001, *p* < 0.001, respectively) (Figure 4C,D). Decreases in the expression levels of fibronectin were observed in the 10 and 20 mg/mL TSE-treated groups compared with the vehicle-treated TGF-β2 group (*p* < 0.001, *p* < 0.001, respectively) (Figure 4E,F). Decreases in the expression levels of collagen I were observed in the 10 and 20 mg/mL TSE-treated groups compared with the vehicle-treated TGF-β2 group (*p* < 0.001, *p* < 0.001, respectively) (Figure 4E,F).

### 3.6. TSE Inhibits the Nuclear Translocation of SMADs

**Figure 5:**
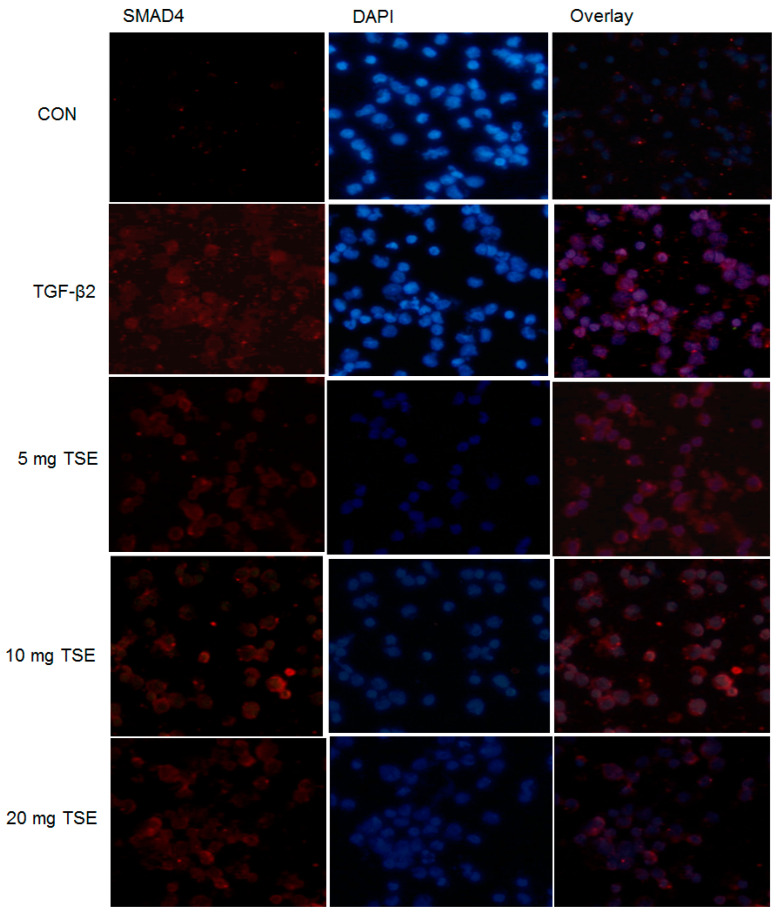
Effects of TSE on epithelial–mesenchymal translocation of SMAD4 using confocal microscopy. The mesenchymal phenotypic marker SMAD4 (red). The nuclei were stained with DAPI (blue). Magnification, ×200.

**Figure 6:**
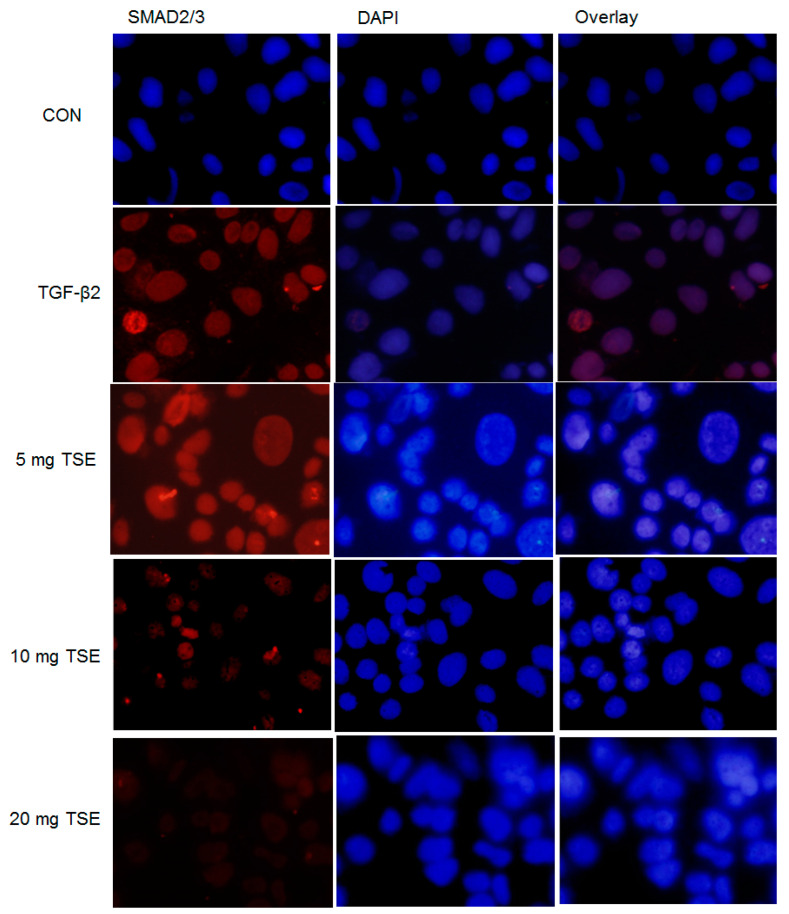
Effects of TSE on epithelial–mesenchymal translocation of SMAD2/3 in cells using confocal microscopy. The mesenchymal phenotypic marker SMAD2/3 (red) in cells. The nuclei were stained with DAPI (blue). Magnification, ×200.

By immunofluorescence assay, the expression levels of SMAD2/3 and SMAD4 protein in SRA01/04 cells were scanned and examined. SMAD2/3 and SMAD4 may be expressed within the cells and nearly in the cytoplasm. As shown in Figure 5 and Figure 6, the nuclear SMAD2/3 or SMAD4 staining in the control group (in the absence of TGF-β2) in SRA01/04 cells was very weak. These results were evaluated under confocal microscopy. Following treatment with 5, 10, or 20 mg/mL TSE, nuclear SMAD2/3 and nuclear SMAD4 expression was decreased in the SRA01/04 cell line compared with the control under microscopy at 200× magnification, and the suppressive activity of 20 mg/mL TSE was the most effective (Figure 5 and Figure 6). Thus, these findings demonstrated that TSE displayed a suppressive effect on SMAD2/3 or SMAD4 translocation to the nucleus in SRA01/04 cells.

### 3.7. Effects of TSE on the Protein Expression of p- JUN/JUN, p-ERK1/2/ERK1/2, p-p38/p38, p-Akt (Ser^473^)/t-Akt, and p-Akt (Thr^308^)/t-Akt in SRA01/04 Cells

**Figure 7:**
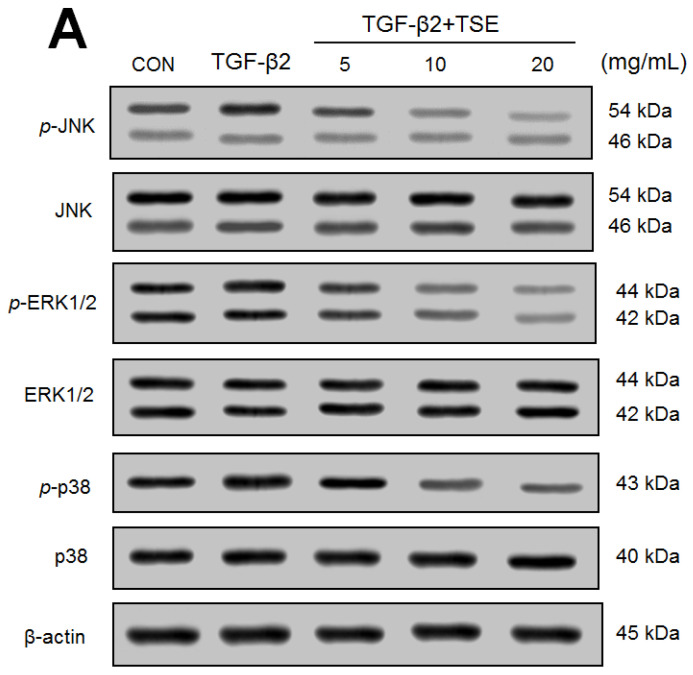

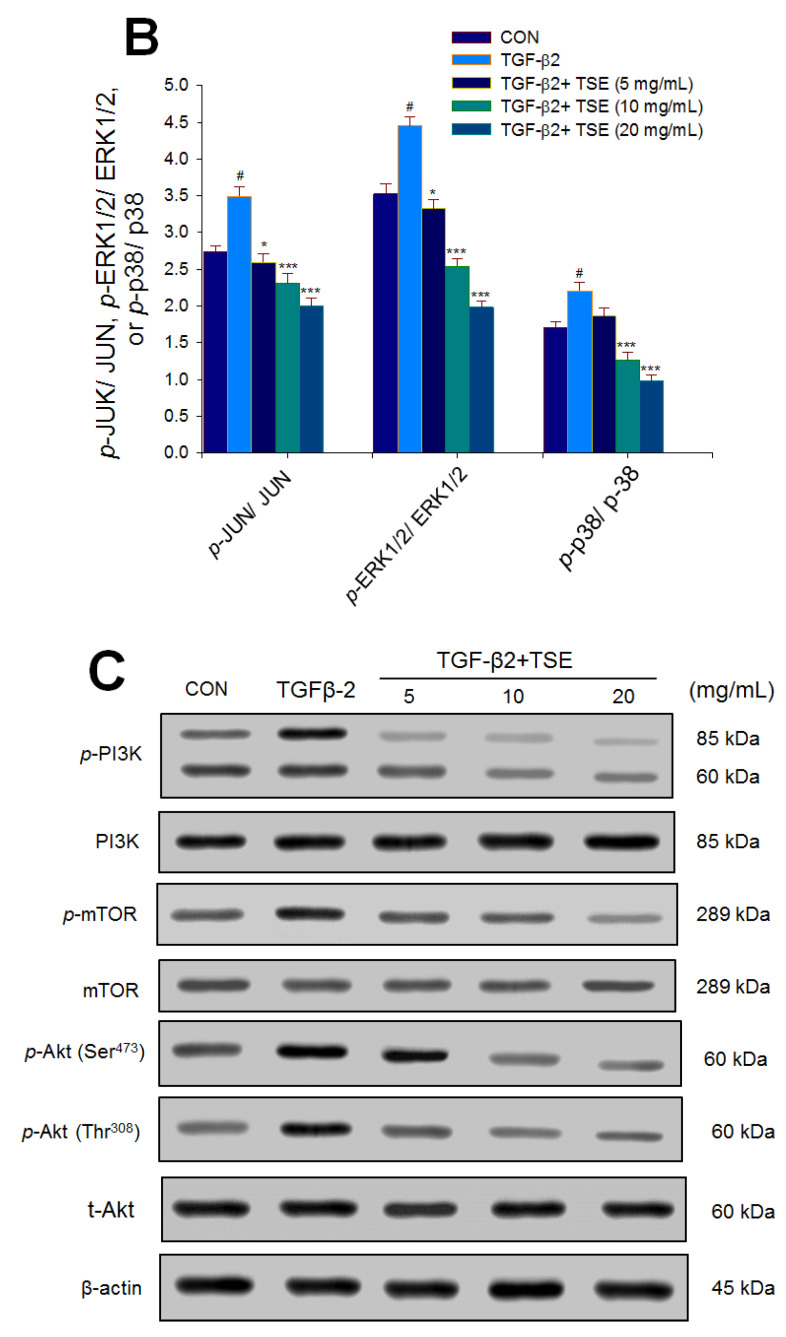

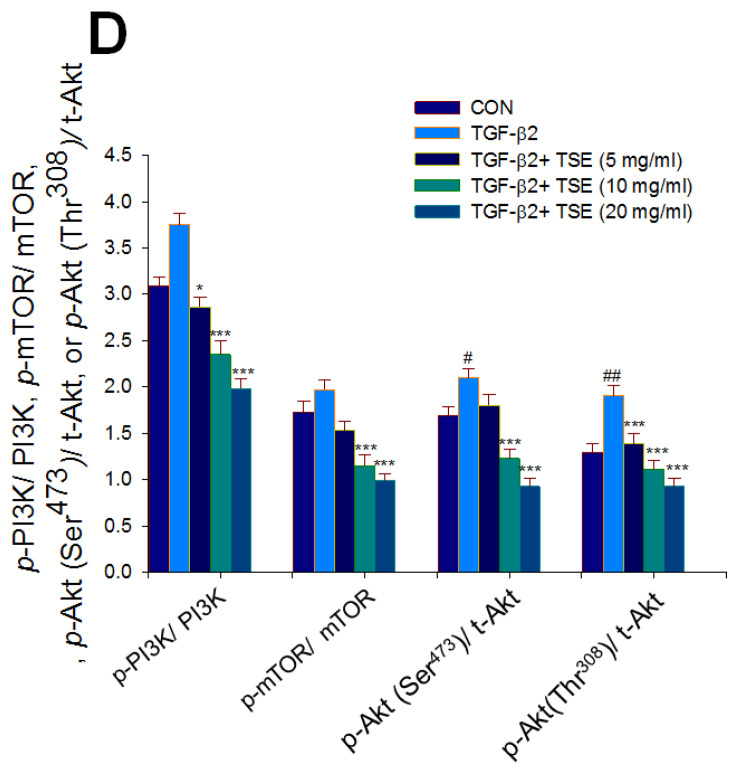
Effects of TSE on expression levels of *p*- JUN/ JUN, *p*-ERK1/2/ ERK1/2, *p*-p38/ p38, *p*-PI3K/ PI3K, *p*-mTOR/ mTOR, *p*-Akt (Ser^473^)/ t-Akt, and *p*-Akt (Thr^308^)/ t-Akt in SRA01/04 cells by Western blot. (**A**) representative image, and (**B**) quantification of the *p*- JUN/ JUN, *p*-ERK1/2/ ERK1/2, and *p*-p38/ p38 to β-actin. Protein was separated by 12% SDS-PAGE. (**C**) representative image, and (**D**) quantification of *p*-PI3K/ PI3K, *p*-mTOR/ mTOR, *p*-Akt (Ser^473^)/ t-Akt, and *p*-Akt (Thr^308^)/ t-Akt to β-actin. Protein was separated by 12% SDS-PAGE. (**C**) representative image, and (**D**) quantification of *p*-PI3K, *p*-mTOR, *p*-Akt (Ser^473^), and *p*-Akt (Thr^308^) to β-actin. ^#^ *p* < 0.05 or ^##^ *p* < 0.01 in comparison to control cells. * *p* < 0.05 or *** *p* < 0.001 in comparison to TGF-β2-induced control cells. Data are the means ±SE (n = 3).

The expression levels of p- JUN/JUN, p-ERK1/2/ERK1/2, p-p38/ p38, p-Akt (Ser^473^)/t-Akt, and *p*-Akt (Thr^308^)/t-Akt were significantly increased in TGF-β2-induced cells compared with the CON cells (*p* < 0.05, *p* < 0.05, *p* < 0.05, *p* < 0.05, *p* < 0.01, respectively) (Figure 7A–D). A decrease in *p*-JUN/JUN and *p*-ERK1/2/ ERK1/2 expression was found in the 5, 10 and 20 mg/mL TSE-treated groups compared to the vehicle-treated TGF-β2 group (Figure 7A,B). A decrease in *p*-p38/ p38 expression was found in the 10 and 20 mg/mL TSE-treated groups compared to the vehicle-treated TGF-β2 group (*p* < 0.001, *p* < 0.001, respectively) (Figure 7A,B). A decrease in *p*-PI3K/ PI3K and *p*-Akt (Thr^308^)/ t-Akt expression was found in the 5, 10, and 20 mg/mL TSE-treated groups compared to the vehicle-treated TGF-β2 group (Figure 7C,D). A decrease in *p*-mTOR/ mTOR and p-Akt (Ser^473^)/ t-Akt expression was found in the 10 and 20 mg/mL TSE-treated groups compared to the vehicle-treated TGF-β2 group (Figure 7C,D).

## 4. Discussion

Cataract is one of the major elderly topics worldwide. Posterior capsular opacification (PCO) is a familiar complicative issue following cataract surgery. Currently, there are various means of management and prevention of PCO, including surgical advances, IOL, anti-metabolic agents, and biological targets. Targeting TGF-β signaling is a modern method for developing a novel therapeutic agent [27]. Therefore, we hypothesized that there are therapeutic interventions with no toxic side effects and low cost that block the TGFβ-2 and SMAD pathways, leading to antifibrotic effects and contributing to the prevention of PCO. Consistently, we found that TSE decreased the mRNA and protein expression levels of TGF-β2, SMAD3, and SMAD4; moreover, TSE inhibited the nuclear translocation of SMAD2/3 and SMAD4 in TGF-β2-induced fibrotic cells, and this vital cell was associated with the management of PCO. Therefore, it is possible that TSE might be a novel and prospective agent against PCO.

Pirfenidone (PFD) is an anti-inflammatory, antioxidant, and antifibrotic agent used in animal studies [9,18,28]. Pirfenidone has been demonstrated to inhibit the growth of orbital fibroblasts at concentrations of 1.9 mg/mL in patients with thyroid-associated ophthalmopathy [10,29].

These five compounds (2,4-dihydro-3H-pyrazol-3-one (DHPO) derivatives) were synthesized by our team with a core structure similar to that of PFD. Our findings show that the activity of these five compounds displays almost similar activities at concentrations of 10 μg/mL but more efficacy than PFD (at concentrations of 0.25 mg/mL) on the expression levels of TGF-β2, SMAD3, or SMAD4, implying that these five compounds display antifibrotic activity approximately 25 times that of PFD in SRA01/04 cells. Based on the results of protein expression of TGF-β2, we selected the most efficient compound and examined the protective activity and its underlying molecular mechanism of TSE.

In this study, we investigated whether TSE displayed targeting TGFβ signaling to provide new insights for developing a novel therapeutic intervention. Our data showed that in the MTT test, 10 μg/mL TSE exhibited no toxic effect in SRA01/04 cells (Figure 3A). Moreover, TSE displayed no LDH activity discharging from the injured cells without harming these cells (Figure 3B). However, this study demonstrates for the first time the beneficial effects of anti-PCO in TGF-β2-induced SRA01/04 cells, thereby producing evidence of the role of TSE’s anti-fibrotic effects by inhibition of TGF-β2-SMAD and non-SMAD signaling pathways.

Previous works from laboratories showed that the SRA01/04 model has been widely used [10,30] and is a key measure of the possible reasons for cataract development. Therefore, for further analysis, we used this cell line to investigate the potential anti-PCO activity of TSE.

Pirfenidone exerted its antifibrotic effect in the following cases. A recent study reported that pirfenidone decreases the levels of mRNA and TGF-β expression in animal models of lung fibrosis [30]. In addition, pirfenidone was found to downregulate the mRNA levels of TGF-β1 in a rodent hepatic fibrosis study [31]. Increasing evidence shows that pirfenidone not only inhibits collagen but also decreases TGF-β expression in a renal fibrosis model [32,33,34].

A recent publication confirmed many of our findings with a reduction in the nuclear accumulation and/or translocation of SMAD2/3 and SMAD4 following treatment with pirfenidone [10]. A recent study found that pirfenidone inhibits TGF-β signaling with suppression of active SMAD2/3 complexes in the nuclei accumulation but does not complete SMAD2/3 phosphorylation [35]. Therefore, there is a possibility that TSE acts as PFD and reduces the nuclear accumulation and/or translocation of SMAD2/3 and SMAD4. In addition to the abovementioned findings, all of these findings point to at least one pathway that results in TSE inhibiting TGF-β2-induced fibrogenic LECs with a decrease in the expression levels of collagen I and fibronectin and reducing the mRNA and expression levels of TGF-β2, SMAD3, or SMAD4, as well as a decrease in the expression levels of p-ERK p42/44 and p-JUN, resulting in the prevention of PCO occurrence.

In addition to SMAD2/3/4 nuclear translocation, there are many signaling pathways involved in the TGF-β receptor-induced response. TGF-β can induce EMT by direct phosphorylation of SMAD2/3 or activation of non-SMAD pathways, such as mitogen-activated protein kinase (MAP) kinase (MAPK; *p*-38, ERK1/2, JUN), mTOR, or PI3K/Akt, thus contributing to the inhibition of epithelial target genes and mesenchymal marker activation [36,37,38]. The additional exact molecular mechanisms involved in the suppression of the TGF-β2-SMAD signaling pathway by TSE remain to be elucidated. Further study will be needed to investigate other potential mechanisms, including activation of the non-SMAD signaling pathway (such as Hippo/YAP, β-catenin/Wnt, proKin, or Rho GTPase) of TSE for suppressive activity in TGF-β2-induced fibrogenic effects in LECs. Because SRA01/04 cells were used in this study, we speculate that TSE might be another candidate for protection against PCO.

## 5. Conclusions

**Figure 8:**
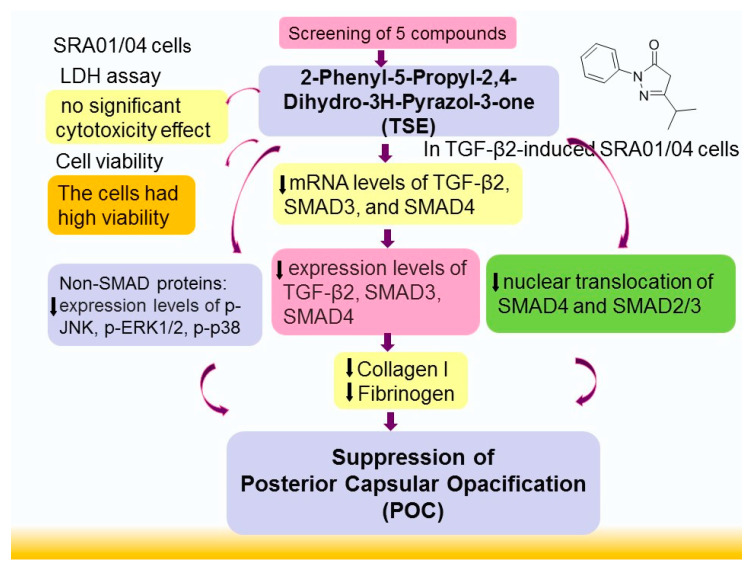
Graphic abstract of 2-phenyl-5-propyl-2,4-dihydro-3H-pyrazol-3-one (TSE).

In summary (Figure 8), the present study demonstrated that TSE has therapeutic potential in treating PCO associated with its antifibrotic activity in a TGF-β2-induced fibrogenesis SRA01/04 cell model. The major target of TSE is the decrease in the expression levels of numerous target genes involved in the TGF-β2/SMAD and non-SMAD signaling pathways and the inhibition of nuclear translocation of SMADs. TSE treatment decreased the mRNA or expression levels of TGF-β2, SMAD3, or SMAD4, with consequent reduced accumulation of SMADs in the nuclei and translocation, as well as decreased non-SMAD pathway gene expression levels of *p*-JUN/ JUN, *p*-ERK1/2/ ERK1/2, *p*-p38/ p38, *p*-PI3K/ PI3K, *p*-mTOR/ mTOR, *p*-Akt (Ser^473^)/ t-Akt, and *p*-Akt (Thr^308^)/ t-Akt in TGF-β2-induced fibrogenic cells. Furthermore, TSE decreased the expression levels of collagen I and fibronectin. The overall effect of TSE is to decrease TGF-β2-induced fibrogenesis in cells, thus resulting in antifibrotic activity with the potential to prevent PCO occurrence.

## Data Availability

All data used to support the findings of this study are available from the corresponding author, Chun-Ching Shih, upon reasonable request. Corresponding author’s email: ccshih@ctust.edu.tw.

## References

[1] Symonds J.G., Lovicu F.J., Chamberlain C.G. (2006). Posterior capsule opacification-like changes in rat lens explants cultured with TGFbeta and FGF: Effects of cell coverage and reginal differences. Exp. Eye Res..

[2] Wormstone I.M., Tamiya S., Anderson I., Duncan G. (2002). TGF-β2-induced matrix modification and cell transdifferentiation in the human lens capsular bag. Investig. Ophthalmol. Vis. Sci..

[3] Wormstone I.M., Wormstone Y.M., Smith A.J.O., Eldred J.A. (2020). Posterior capsule opacification: What’s in the bag?. Prog. Retin. Eye Res..

[4] Johnson W.J., Magrath G.N., Poole Perry L.J. (2019). Rapid anterior capsule contraction after femtosecond laser-assisted cataract surgery in a patient with retinitis pigmentosa. JCRS Online Case Rep..

[5] Wang M., Zhang J.J., Jackson T.L., Sun X., Wu W., Marshall J. (2007). Safety and efficacy of intracapsular tranilast microspheres in experimental posterior capsule opacification. J. Cataract. Refract. Surg..

[6] Pandey S.K., Cochener B., Apple D.J., Colin J., Werner L., Bougaran R., Trivedi R.H., Macky T.A., Tzak A.M. (2002). Intracapsular ring sustained 5-fluorouracil delivery system for the prevention of posterior capsule opacification in rabbits: A histological study. J. Cataract. Refract. Surg..

[7] Yu S., Lu C., Tang X., Yuan X., Yuan B., Yu Z. (2018). Application of spectral domain optical coherence tomography to objectively evaluate posterior capsular opacity in vivo. J. Ophthalmol..

[8] Buehl W., Findl O. (2008). Intraocular lens design on posterior capsule opacification. J. Cataract. Refract. Surg..

[9] Hazra S., Palui H., Vemuganti G.K. (2012). Comparison of design of intraocular lens versus the material for PCO prevention. Int. J. Ophthalmol..

[10] Yang Y., Ye Y., Lin X., Wu K., Yu M. (2013). Inhibition of pirfenidone on TGF-beta2 induced proliferation, migration and epithelial-mesenchymal transition of human lens epithelial cells line SRA01/04. PLoS ONE.

[11] Wu S., Tong N., Pan L., Jiang X., Guo M., Li H. (2018). Retrospective analyses of potential risk factors for posterior capsule opacification after cataract surgery. J. Ophthalmol..

[12] Lee E.H., Joo C.K. (1999). Role of transforming growth factor-beta in transdifferentiation and fibrosis of lens epithelial cells. Investig. Ophthalmol. Vis. Sci..

[13] Zhang R., Li X., Liu Y., Gao X., Zhu T., Liu L. (2019). Acceleration of bone regeneration in critical-size defect using BMP-9-loaded nHA/ColI/MWCNTs scaffolds seeded with bone marrow mesenchymal stem cells. Biomed. Res. Int..

[14] Dawers L.J., Angell H., Sleeman M., Reddan J.R., Wormstone L.M. (2007). TGFbeta isoform dependent SMAD2/3 kinetics in human lens epithelial cells: A cellomics analysis. Exp. Eye Res..

[15] Saika S., Yamanaka O., Okada Y., Tanaka S.-I., Miyamoto T., Sumioka T., Kitano A., Shirai K., Ikeda K. (2009). TGF beta in fibroproliferative disease in the eye. Front. Biosci..

[16] Saika S., Yamanaka O., Flanders K.C., Okada Y., Miyamoto T., Sumioka T., Shirai K., Kitano A., Miyazaki K., Tanaka S. (2008). Epithelial-mesenchymal transition as a therapeutic target for prevention of ocular tissue fibrosis. Endocr. Metab. Immune Disord. Drug Targets.

[17] Katsuno Y., Lamouille S., Derynck R. (2013). TGF-β signaling and epithelial-mesenchymal transition in cancer progression. Curr. Opin. Oncol..

[18] Hilberg O., Simonsen U., Du Bois R., Bendstrup E. (2012). Pirfenidone: Significant treatment effects in idiopathic pulmonary fibrosis. Clin. Respir. J..

[19] Huang Y.Y., Lin H.C., Cheng K.M., Su W.N., Sung K.C., Lin T.P., Huang J.J., Lin S.K., Wong F.F. (2009). Efficient debromination of 5-pyrazolones and 5-hydroxypyrazoles by N-bromobenzamide. Tetrahedron.

[20] Pawlowski P., Reszec J., Eckstein A., Johnson K., Grzybowski A., Chyczewski J., Mysliwiec L. (2014). Markers of inflammation and fibrosis in the orbital fat/connective tissue of patients with Graves’ orbitopathy: Clinical implications. Mediat. Inflamm..

[21] Ibaraki N., Chen S.C., Lin L.R., Okamoto H., Pipas J.M., Reddy V.N. (1998). Human lens epithelial cell line. Exp. Eye Res..

[22] Li D.D., Liu Y., Yuan R.R., Yu T., Yang B., Wang W.Y. (2019). Antifibrotic effect of pirfenidone on orbital fibroblasts in patients with thyroid-associated ophthalmopathy and its mechanisms. Zhonghua Nei Ke Za Zhi.

[23] Olmos-Zuňiga J.R., Baltazares-Lipp M., Hernándéz-Jimenez C., Victoria R.J., Gaxiola-Gaxiola M., Silva-Martinez M., Iňiguez-Garcia M.A., González-González A.I., Vázquez-Minero J.C., Luna-Flores A. (2020). Treatment with hyaluronic acid and collagen-polyvinylpyrrolidone improves extracellular matrix assembly for scarring after tracheal resection. Biomed. Res. Int..

[24] Mancini R., Marucci L., Benedertti A., Jezeque A.-M., Oralndi F. (1994). Immunohistochemical analysis of S-phase cells in normal human and rat liver by PC10 monoclonal antibody. Liver Int..

[25] Shimizu T., Kuroda T., Hata S., Fukagawa M., Margolin S.B., Kurokawa K. (1998). Pirfenidone improves renal function and fibrosis in the post-obstructed kidney. Kidney Int..

[26] Everett I.V.T.H., Olgin J.E. (2007). Atrial fibrosis and the mechanisms of atrial fibrillation. Heart Rhythm.

[27] Shrestha B.N., Haylor J. (2014). Biological pathways and potential targets for prevention and therapy of chronic allograft nephropathy. Biomed. Res. Int..

[28] Choi K., Lee K., Ryu S.W., Im M., Kook K.H. (2012). Pirfenidone inhibits transforming growth factor-β1-induced fibrogenesis by blocking nuclear translocation of Smads in human retinal pigment epithelial cell line ARPE-19. Mol. Vis..

[29] Sutrisno S., Sulistyorini C., Manungkalit E.M., Winarsih L., Noorhamdani N., Winarsih S. (2017). The effect of genistein on TGF-β signal, dysregulation of apoptosis, cyclooxygenase-2 pathway, and NF-κB pathway in mice peritoneum of endometriosis model. Middle East Fertil. Soc. J..

[30] Luo K. (2017). Signaling cross talk between TGF-β/ Smad and other signaling pathways. Cold Spring Herb. Perspect. Biol..

[31] Shu D.Y., Butcher E., Saint-Geniez M. (2020). EMT and EndMT: Emerging roles in age-related macular degeneration. Int. J. Mol. Sci..

[32] Longo C.M., Higgins P.J. (2019). Molecular biomarkers of Graves’ ophthalmopathy. Exp. Mol. Pathol..

[33] Ye J.-C., Hsu L.-S., Tsai J.-H., Yang H.-L., Hsiao M.-W., Hwang J.-M., Lee C.-J., Liu J.-Y. (2017). MZF-1/Elk-1/PKCα is associated with poor prognosis in patients with hepatocellular carcinoma. J. Cancer.

[34] Wang L., Xu Z., Ling D., Li J., Wang Y., Shan T. (2020). The regulatory role of dietary factors in skeletal muscle development, regeneration and function. Crit. Rev. Food Sci. Nutr..

[35] Atwood J.J., Buck W.R. (2020). Recent literature on bryophytes−123(2). Bryologist.

[36] Lin C.H., Kuo Y.H., Shih C.C. (2014). Effects of Bofu-tsusho-san on diabetes and hyperlipidemia associated with AMP-activated protein kinase and glucose transporter 4 in high-fat-fed mice. Int. J. Mol. Sci..

[37] Rahimova N., Cooke M., Zhang S., Baker M.J., Kazanietz M.G. (2020). The PKC universe keeps expanding: From cancer initiation to metastasis. Adv. Biol. Regul..

[38] Ma X., Lin W., Lin Z., Hao M., Gao X., Zhang Y., Kuang H. (2017). Liraglutide alleviates H_2_O_2_-induced retinal ganglion cells injury by inhibiting autophagy through mitochondrial pathways. Peptides.

